# Common HLA Alleles Associated with Health, but Not with Facial Attractiveness

**DOI:** 10.1371/journal.pone.0000640

**Published:** 2007-07-25

**Authors:** Vinet Coetzee, Louise Barrett, Jaco M. Greeff, S. Peter Henzi, David I. Perrett, Ahmed A. Wadee

**Affiliations:** 1 Department of Genetics, University of Pretoria, Pretoria, South Africa; 2 Department of Psychology, University of Lethbridge, Lethbridge, Canada; 3 Department of Psychology, University of St. Andrews, St. Andrews, Scotland; 4 Department of Immunology, University of the Witwatersrand, Johannesburg, South Africa; University of Birmingham, United Kingdom

## Abstract

Three adaptive hypotheses have been proposed to explain the link between the human leucocyte antigen (HLA) genes, health measures and facial attractiveness: inbreeding avoidance, heterozygote advantage and frequency-dependent selection. This paper reports findings that support a new hypothesis relating HLA to health. We suggest a new method to quantify the level of heterozygosity. HLA heterozygosity did not significantly predict health measures in women, but allele frequency did. Women with more common HLA alleles reported fewer cold and flu bouts per year, fewer illnesses in the previous year and rated themselves healthier than women with rare alleles. To our knowledge, this is the first study to show a positive correlation between HLA allele frequency and general health measures. We propose that certain common HLA alleles confer resistance to prevalent pathogens. Nevertheless, neither HLA heterozygosity nor allele frequency significantly predicted how healthy or attractive men rated the female volunteers. Three non-mutually exclusive explanations are put forward to explain this finding.

## Introduction

Evolutionary theory predicts that individuals should choose partners that will increase the number and quality of offspring produced, and that such choices will be based on phenotypic characteristics associated with the ability to supply partners and offspring with direct and indirect benefits. Direct benefits include the ability of the potential partner to provide resources, such as food, shelter, parental care and protection from parasite due to his own reduced parasite load [1]. These benefits have an immediate effect on reproductive success [1]. An individual also benefit indirectly from choosing a partner with traits that indicate their “high quality”. Hamilton and Zuk [2] proposed that these “high quality” traits or “good genes” could relate to disease resistance in the potential partner. Disease resistance can be judged by a wide array of traits including bright plumage, symmetry or even a vigorous courtship display. If a male cannot cope with the continuous onslaught of pathogens his sexual signals would deteriorate and the display would be somewhat less thrilling. These genes will be passed on to the offspring reducing their inherent resistance to disease.

Conventionally females are considered the choosier sex, because of their excessive reproductive investment in offspring [3]. However, humans have a resource-based mating system and men invest substantially in offspring, especially since offspring only become independent relatively late in life. Women, therefore, prefer men that can shoulder such a large resource-based burden, and these men in turn are choosier about the women they select as partners [3]–[5]. Thus, in humans at least, both sexes prefer partners that can provide them with direct and indirect benefits. Though males traditionally provide most of the direct benefits in the form of food, shelter and protection against predators and parasites [6], both sexes benefit indirectly from choosing “high genetic quality” partners, as these “good genes” are inherited by offspring, and confer survival and/or reproductive advantages.

But what exactly is “genetic quality”? According to Neff&Pitcher [7] “genetic quality” can be defined as the sum of two components: additive genetic effects or “good genes” and non-additive genetic effects or “compatible genes”. Specific alleles that increase fitness independently of the rest of the genome contribute to additive genetic benefits, while heterozygote advantage and epitasis contribute to non-additive genetic benefits. Both additive and non-additive genetic effects are considered important determinants of “genetic quality” and by implication, of mate choice (for a review see [7]).

One of the best-studied genetic based mating systems in vertebrates is the major histocompatibility complex (MHC). The MHC plays an essential role in immune response, where it serves as antigen presenting molecules [8], [9]. Several previous studies have linked the MHC to mating preferences in mice [10]–[12], savannah sparrows [13], Atlantic salmon [14] and humans [15], although there are also studies that show no such effects (Soay sheep [16]; humans [17]–[19]).

According to past research MHC based mating preferences are primarily driven by inbreeding avoidance, heterozygote advantage and frequency dependent selection (for a review see [20]). Since specific rare alleles confer fitness benefits under frequency-dependent selection, and such alleles would still confer these benefits without the contribution of any other alleles, frequency-dependent selection is driven by additive genetic effects.

In a recent study, Roberts *et al.* [21] linked a form of “genetic quality”, HLA heterozygosity, to a sexually selected trait, facial attractiveness. They showed that HLA heterozygous men are perceived healthier and more attractive than more HLA homozygous men. A previous study by Thornhill *et al.* [22] did not find this link between HLA heterozygosity and facial attractiveness among males. Moreover, a more recent study [23] did not find a significant relationship between facial attractiveness and health-history measures. The preference for HLA heterozygosity observed by Roberts *et al.* [21] could be due to two, non-mutually exclusive, HLA based mating preferences. First, individuals might prefer heterozygous mates because such individuals will confer direct benefits in the form of increased health and therefore better resource-holding potential, or indirect benefits in the form of more heterozygous offspring [24]. Secondly, the preference for heterozygosity could be a by-product of a preference for “rare alleles”, because heterozygous individuals are more likely to possess such rare alleles than homozygous individuals [22]. Our objectives were to (a) investigate the health benefits associated with the two pathogen-driven selection pressures: frequency dependent selection and heterozygote advantage and (b) the role of heterozygote advantage and frequency dependent selection, in predicting HLA based facial preferences. We achieved both objectives. Our study differs from previous studies in three important respects. First, we tested male choice of female partners, rather than female preferences for males. This is because human males also contribute substantially to offspring, and males should therefore be equally choosy about the quality of their mates. To date, male preferences of this kind have been largely ignored in the literature. Second, our sample population group has a much higher pathogen load than previously studied populations [25]. Therefore, one might expect stronger selection by pathogen-driven selective forces (heterozygote advantage, frequency dependent selection and fluctuating selection [26]) in our population than in populations with a lower pathogen load. Third, we also looked at genetic distance between alleles, rather than just heterozygosity *per se*. Since the amount of overlap between HLA molecules (and therefore the genetic distance between these molecules) dictate the extent of the benefit associated with heterozygosity [27], genetic distance will serve as a more sensitive measure of heterozygosity.

## Results

Allele (Table 1) and phenotype frequencies were submitted to http://www.allelefrequencies.net and are available in the category South African Tswana

**Table 1:** HLA-A and HLA-B allele frequencies of the South African Tswana population.

Allele	Allele frequency	Allele	Allele frequency
**HLA-A**		**HLA-B**	
A*30	0.159	B*58	0.220
A*02	0.146	B*15	0.159
A*29	0.122	B*44	0.116 ^♦^
A*68	0.122	B*45	0.110 ^♦♦^
A*03	0.085	B*42	0.085
A*23	0.085	B*13	0.061
A*34	0.073	B*14	0.061
A*43	0.049	B*18	0.049
A*66	0.037	B*08	0.037
A*26	0.024	B*81	0.037
A*01	0.012	B*07	0.024
A*31	0.012	B*50	0.018 ^♦^
A*32	0.012	B*40	0.012
A*36	0.012	B*35	0.006 ^♦^
A*80	0.012	B*53	0.006 ^♦^

Alleles are arranged from most common to least common in each locus. ^♦^ = Three individuals showed ambiguous allele classifications for HLA-B (44&45/44&50; 7&35/7&53; 13&44/13&45). In such cases both ambiguous alleles were awarded a half score).

### (a) *HLA heterozygosity, health and facial attractiveness*

We found no significant difference in any of the health or attractiveness measures between the heterozygous and homozygous groups. Heterozygous females did not rate themselves as significantly healthier than homozygotes (t_39_ = −0.323, p = 0.748), nor did they report fewer illnesses in the previous year (t_38_ = 0.028, p = 0.977) or cold and flu bouts per year (t_37_ = 0.087, p = 0.931; Discrepancies in degrees of freedom are due to omitted answers on illnesses in the previous year [1 participant] and number of cold and flu bouts per year [2 participants]). Male raters also did not rate heterozygous females as being significantly healthier (t_39_ = −0.130, p = 0.897) or more attractive (t_39_ = 1.186, p = 0.243).

**Figure 1:**
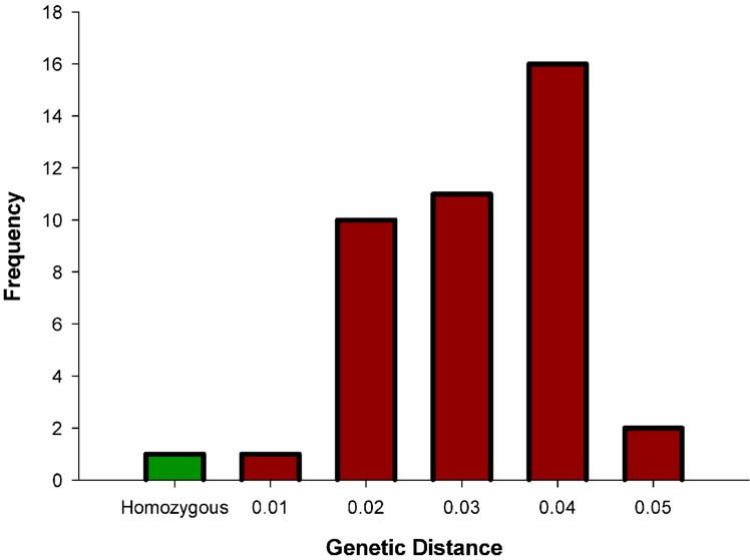
Frequency of genetic distances averaged over HLA-A and HLA-B. Homozygotes are indicated in green and heterozygotes in red.

We calculated combined genetic distances for both HLA loci as a second measure of HLA heterozygosity (Figure 1). These were used to test associations between HLA genetic distance (as a continuous measure of HLA heterozygosity) and health and attractiveness measures. Genetic distance did not significantly predict self-rated health (F_1,37_ = 1.229, R^2^ = 0.032, p = 0.275), number of illnesses in the previous year (F_1,35_ = 0.356, R^2^ = 0.010; p = 0.555) or number of cold and flu bouts per year (F_1,34_ = 0.242, R^2^ = 0.007; p = 0.626). In addition, larger genetic distances did not predict how healthy (F_1,38_ = 0.532, R^2^ = 0.014, p = 0.470) or attractive (F_1,37_ = 0.017, R^2^<0.001; p = 0.897) females were rated by the opposite sex.

### (b) *HLA allele frequency, health and facial attractiveness*

**Figure 2:**
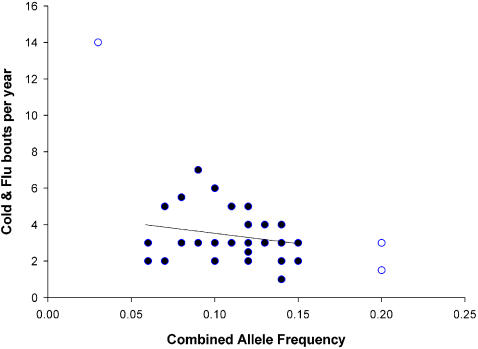
The relationship between combined allele frequency and the number of self reported cold and flu bouts per year. Outliers are indicated as open circles.

**Figure 3:**
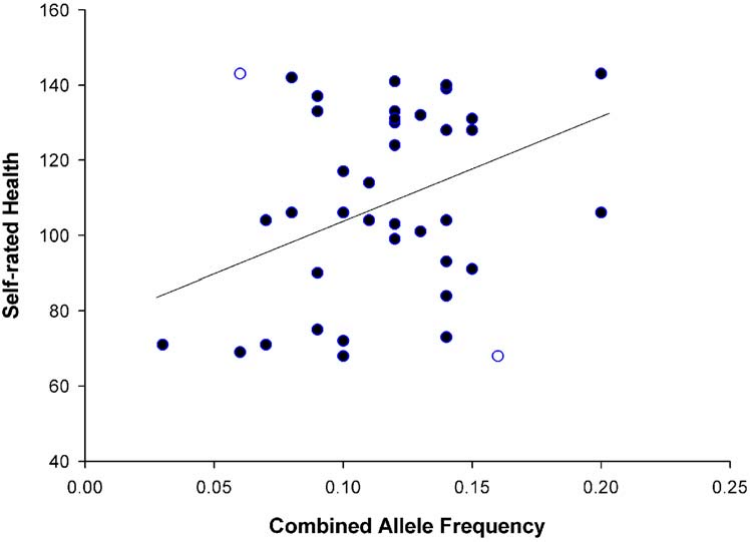
The relationship between combined allele frequency and self-rated health. Outliers are indicated as open circles.

**Figure 4:**
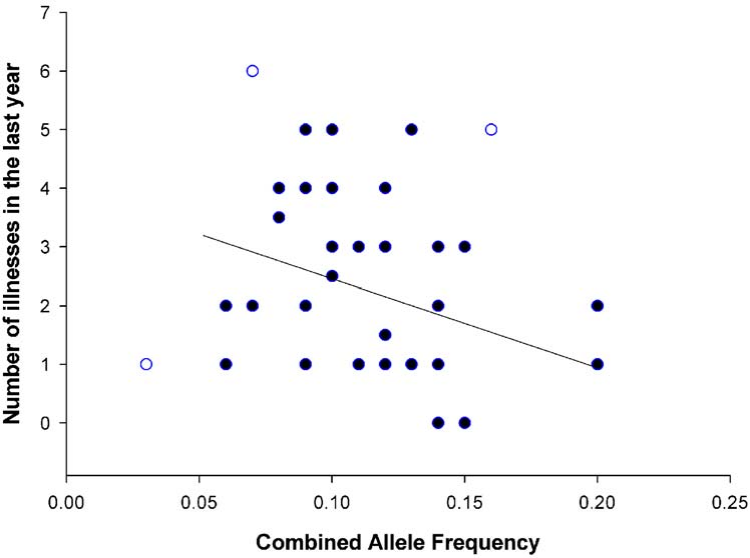
The relationship between combined allele frequency and the number of self reported illnesses in the previous year. Outliers are indicated as open circles.

Combined allele frequency (averaged for HLA-A and HLA-B) significantly predicted the number of cold and flu bouts per year (equation: Cold and flu bouts = 5.536–18.743×allele frequency, F_1,34_ = 5.618, R^2^ = 0.142, p = 0.024; Figure 2), and predicted self-rated health and number of illnesses in the previous year after the removal of influential outliers (Self-rated health: equation: Self-rated health = 74.715+288.08×allele frequency, F_1,37_ = 6.587, R^2^ = 0.151, p = 0.014; Figure 3)(Number of illnesses: equation: Ill previous year = 4.089–16.245×allele frequency, F_1,35_ = 5.014, R^2^ = 0.125, p = 0.032; Figure 4), and not before (Self-rated health: F_1,39_ = 2.221, R^2^ = 0.054, p = 0.144)(Number of illnesses: F_1,38_ = 2.357, R^2^ = 0.058, p = 0.133; Table 2). However, the effect of illnesses per year was robust enough to give a marginally significant (p = 0.06) rank correlation before the removal of outliers. Interestingly, these results show that females with high combined allele frequencies (i.e., those with more common alleles) considered themselves healthier and reported fewer illnesses.

**Table 2:** HLA allele frequency as a predictor of health and attractiveness measures.

Source	Df	F	p	R^2^
Self-rated health				
** Both loci**	**1, 37**	**6.587**	**0.014**	**0.151**
** HLA-A**	**1, 37**	**8.946**	**0.005**	**0.195**
HLA-B	1, 35	1.185	0.284	0.033
Number of times Ill last year				
** Both loci**	**1, 35**	**5.014**	**0.032**	**0.125**
HLA-A	1, 35	3.182	0.083	0.083
HLA-B	1, 35	2.937	0.095	0.077
Cold&Flu's per year				
** Both loci**	**1, 34**	**5.618**	**0.024**	**0.142**
HLA-A	1, 36	0.038	0.847	0.001
** HLA-B**	**1, 34**	**5.829**	**0.021**	**0.146**
Rated health				
Both loci	1, 36	0.175	0.678	0.005
HLA-A	1, 35	1.412	0.243	0.039
HLA-B	1, 36	0.458	0.503	0.013
Rated attractiveness				
Both loci	1, 34	2.421	0.129	0.066
HLA-A	1, 37	0.751	0.392	0.020
HLA-B	1, 36	0.114	0.738	0.003

Significant predictors are indicated in bold.

Conversely, combined allele frequencies did not significantly predict how healthy or attractive females were rated by the opposite sex (Health: F_1,36_ = 0.175, R^2^ = 0.005, p = 0.678; attractiveness: F_1,34_ = 2.421, R^2^ = 0.066, p = 0. 129; Table 2).

**Figure 5:**
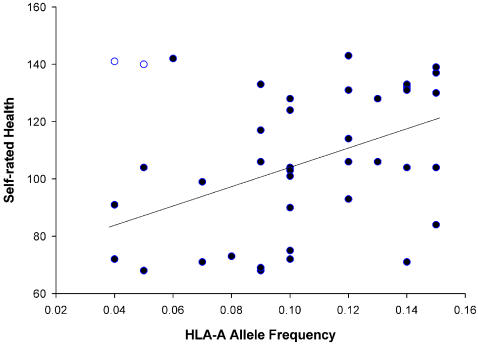
The relationship between HLA-A allele frequency and self–rated health. Outliers are indicated as open circles.

**Figure 6:**
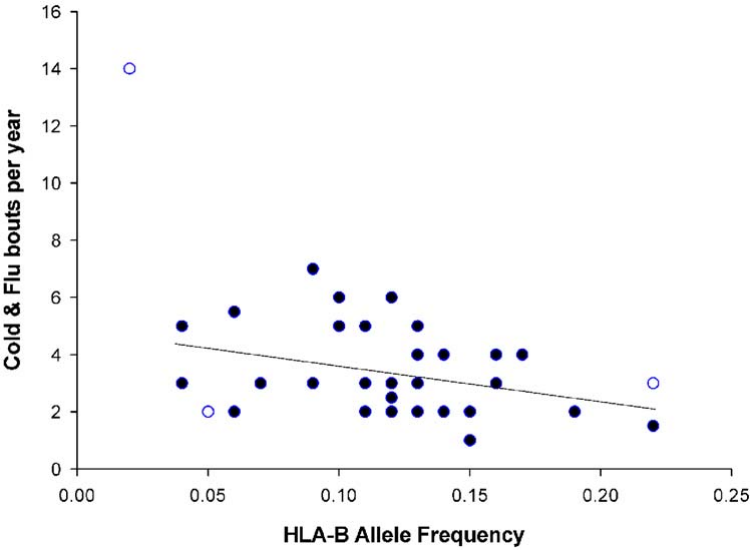
The relationship between HLA-B allele frequency and the number of self reported cold and flu bouts per year. Outliers are indicated as open circles.

Next, we tested if the effect of the combined allele frequency can be explained by either HLA-A or HLA-B. HLA-B allele frequency did not significantly predict self-rated health (F_1,35_ = 1.185, R^2^ = 0.033, p = 0.284), while HLA-A allele frequency significantly predicted self-rated health after the removal of outliers (equation: Self-rated health = 71.347+329.534×allele frequency, F_1,37_ = 8.946, R^2^ = 0.195, p = 0.005; Figure 5) but not before (F_1,39_ = 3.390, R^2^ = 0.080, p = 0.073). On the other hand, HLA-B allele frequency significantly predicted the number of cold and flu bouts per year (equation: Cold&Flu bouts = 5.079–13.686×allele frequency, F_1,34_ = 5.829, R^2^ = 0.146, p = 0.021; Figure 6), while HLA-A allele frequency did not (F_1,36_ = 0.038, R^2^ = 0.001, p = 0.847). Neither HLA-A allele frequency nor HLA-B allele frequency significantly predicted the number of illnesses in the previous year (HLA-A, F_1,35_ = 3.182, R^2^ = 0.083, p = 0.083)(HLA-B, F_1,35_ = 2.937, R^2^ = 0.077, p = 0.095).

On their own, neither HLA-A allele frequency nor HLA-B allele frequency significantly predicted health or attractiveness ratings by males (Health: HLA-A: F_1,35_ = 1.412, R^2^ = 0.039, p = 0.243; HLA-B: F_1,36_ = 0.458, R^2^ = 0.013, p = 0.503; attractiveness: HLA-A: F_1,37_ = 0.751, R^2^ = 0.020, p = 0.392; HLA-B: F_1,36_ = 0.114, R^2^ = 0.003, p = 0.738).

## Discussion

Our results show that women with more common HLA class I alleles rated themselves healthier and reported fewer illnesses, although the class I loci responsible for the beneficial effects differed between health measures. To our knowledge, this is the first study to directly report a positive correlation between HLA allele frequency and general health measures. However, HLA heterozygous women do not report that they are healthier than more homozygous women, nor do they report fewer illnesses. This was true for both the categorical measure of heterozygosity and genetic distance as a measure of heterozygosity. This lack of significance could potentially be due to a small sample size and the consequent lack of statistical power.

One plausible explanation for this novel finding is that, contrary to what might normally be expected, certain common alleles increase an individual's ability to resist specific pathogens. Several previous studies have shown a correlation between specific HLA class I alleles and resistance to chronic Hepatitis B [28], malaria [29] and delayed progression to AIDS [30]. In all three studies, resistance was conferred by alleles that were common in the respective study populations. These findings cannot explain the high allele diversity at HLA loci, which may be driven by less frequent diseases or by inbreeding avoidance. Although we found no evidence for heterozygote advantage, this does not mean that heterozygote advantage cannot drive pathogen-related selection. Our results show only that the benefit associated with the different adaptive hypotheses is likely to be context-specific. HLA heterozygote advantage is expected to be less beneficial in a situation where a few common pathogens cause most ailments. In African populations, most of the infectious diseases are caused by a few common pathogens [31]. In such a scenario, one might expect an exceptional health benefit from alleles that confer resistance to these major pathogens. HLA heterozygosity undoubtedly contributes to general resistance to pathogens, but the specific alleles that confer specific resistance provide more protection under these conditions.

Though women with more common alleles reported better health, men did not rate them as appearing significantly healthier or more attractive. It is unlikely that there are direct allele-specific facial cues to health, and even more unlikely that such cues can be reliably recognised. However, one might be able to pick up a correlation between rated and reported health in a larger study population due to the indirect effects of fitter alleles. Individuals sporting fitter alleles could for instance have increased symmetry or reduced scarring due to their superior immune system. Men also did not rate HLA heterozygous women significantly healthier or more attractive. Heterozygosity may not be an important measure of fitness for the Tswana population who are decidedly inbred because of a high incidence of consanguineous (first cousin) marriages [32]. Regular inbreeding leads to the purging of deleterious recessive alleles. This may be an important difference between Roberts *et al*. [21] study and ours. Furthermore, this study focused on female subjects, whereas previous work has looked at males. Despite male resource based contributions to offspring, selection on males may not have been strong enough for them to detect more subtle individual variations in attractiveness, as opposed to more obvious features such as facial neoteny, averageness and symmetry. In conclusion, we have found evidence for a relationship between HLA and health in a South African population. Allele frequency positively predicts health, presumably through specific common alleles that confer resistance to infectious pathogens. Future research will benefit from including pathogens that impose a greater fitness cost than those pathogens tested here. However, cues to health were not readily recognised in facial features, and healthier individuals are therefore not rated to be either healthier or more attractive.

## Materials and Methods

This project was cleared by the ethics committees of the University of Pretoria (Text S1) and the University of the Witwatersrand (Text S2).

### (a) *Sample group*

Our sample group consisted of 59 female volunteers (age, Mean = 19.8, S.D. = 1.56, Range = 18–26). All participants identified themselves as Tswana, the most abundant Bantu-speaking ethnic group at the University of Pretoria (V. Coetzee, unpublished data). Each volunteer signed a subject information and consent form before taking part in the study (Text S3).

### (b) *Data collection*

Participants completed a questionnaire, providing information on their sex, age, hormonal contraceptive use, sexual preference, self-rated health, number of illnesses in the previous year and the number of cold and flu bouts per year (Text S4). Apart from two participants, no one reported hormonal contraceptive use in the previous year. Three participants reported being homosexual.

Buccal cells were collected by scraping the inside of the mouth for a minimum of ten strokes with a sterile nylon bristle cytology brush. Brush tips were then stored in 300 µl cell lysis solution and kept at room temperature for a maximum of 36 hours, well within the recommended storage period.

All participants were photographed in full colour with a Sony Cybershot DSC P72 (default settings with 3.1 Mega pixels fine, Soft light flash) under standard lighting conditions. Participants were asked to maintain a neutral expression and were seated at a fixed distance from the camera. Two photographs were taken of each participant and the best one used for analyses. Slight lateral tilting of individual faces was corrected by rotation around the facial midline using vertical guidelines and cropped 5cm from each side to standardize size in Coral Photo-Paint v. 10 (Coral Corporation, Ontario, Canada). Next, faces were masked to eliminate confounding factors (e.g., hairstyles) in Coral Knockout v. 1.5 (Coral Corporation, Ontario, Canada). All images were saved in 24-bit RGB colour at 72 dpi.

These masked photographs were used to compile 24 full colour presentations consisting of 5 female photographs each. Presentations were rated by 59 Tswana male (age, Mean = 21.1, S.D. = 2.11, Range = 18–26) participants from the University of the Witwatersrand, to minimise the probability of individuals either recognising or being acquainted with the female participants. Raters assessed opposite sex individuals for attractiveness, perceived health and they also indicated whether or not they knew the rated individual (Text S4). Raters indicated rated attractiveness and perceived health on a continuous line (142 mm), with markings to indicate the ends, centre and quarter points. They were instructed to place a clear mark across the line to indicate their rating for each individual. Individuals that recognised any of the rated individuals were given another presentation set to rate. Rated attractiveness and health ratings were averaged for each rated individual to yield an index of facial attractiveness and health respectively. All volunteers were provided with lunch in return for participation.

### (c) *HLA typing*

We focused specifically on only two HLA loci, HLA-A and HLA-B because these loci are important in all nucleated cells, not just certain cells of the immune system. Genomic DNA was extracted with the Puregene® DNA Purification kit (Gentra Systems, Inc., Minneapolis, USA), according to the manufacturers recommended protocol. After extraction, DNA was resuspended in 20 µl low TE (10 mM Tris, 0.1 mM EDTA). Twenty eight samples with a total DNA content >480 ng were typed for HLA-A and HLA-B using the Dynal RELI™ SSO typing kit (Invitrogen Corporation, California, USA) according to manufacturers instructions. An additional 13 samples (total DNA content >200 ng) were typed with the INNO-LiPA HLA-A/B kits (Innogenetics group, Gent, Belgium), as this method requires less total DNA. Collectively, these methods resolved allele types on the two-digit allele group level for 41 participants. Three individuals showed ambiguous allele classifications for HLA-B, such that one of the HLA-B alleles could belong to one of two allele groups.

To test the first hypothesis that HLA heterozygosity positively influences health and facial attractiveness, we calculated two measures of heterozygosity. The first measure, as previously defined by Roberts *et al.* [21], was the number of alleles shared at both loci. Individuals that were heterozygous at both loci were grouped as heterozygous, while individuals that were homozygous at one or both loci were grouped as homozygous. The three ambiguous allele classifications were all heterozygous for HLA-B and were grouped accordingly.

For our second measure of heterozygosity, we calculated the genetic distance between the two alleles of each locus and averaged the value over both loci. Genetic distance should serve as a more sensitive and continuous measure of heterozygosity than the normal categorical heterozygosity and homozygosity. To calculate genetic distance, we downloaded sequence data for all alleles (4-digit) within a specific allele group (2-digit) from the IMGT/HLA sequence database [33]. We did not download null alleles, unconfirmed alleles and alleles that were shorter than the bulk of the sequence data (HLA-A<1098 bp, HLA-B<1089 bp). Homozygous individuals were assumed to have 4-digit alleles identical by descent and therefore a distance of 0. Alleles were aligned with Clustal W [34] available in BioEdit Sequence Alignment editor v.7.0.5.2 [35] and the genetic distance calculated using DNADist v.3.5c also available in BioEdit Sequence Alignment Editor v.7.0.5.2 as the average distance between all 4-digit alleles within a 2-digit allele group. Separate general linear models (GLM) were fitted for each dependent variable and both measures of heterozygosity.

To test the second hypothesis that rare alleles are positively correlated with health measures, we calculated the allele frequency of HLA-A and HLA-B alleles in our sample group. Each allele frequency was calculated as twice the number of homozygous genotypes with that particular allele, plus the number of heterozygous genotypes with that particular allele, divided by two times the total number of individuals in the sample population [36]. Allele frequencies were averaged across both loci to produce a combined allele frequency. Separate GLM analyses were fitted for each measure of health and facial attractiveness.

All analyses were performed in SPSS version 13.0 (Chicago, USA), alpha was set at 0.05 and all reported cases are two tailed. In each analysis, we calculated Cook's D [37] to identify influential outliers with a critical value of Cook's D>0.11, as determined by Cook's D = 4/n, where n = the number of cases. Once identified, influential outliers were removed from analyses. We report only results obtained after the removal of influential outliers, unless there was a difference in statistical significance, in which case we report both results.

## Supporting Information

Text S1 Ethical approval-University of Pretoria.
(1.22 MB JPG)

Text S2 Ethical approval-University of the Witwatersrand.
(1.77 MB JPG)

Text S3 Subject information and consent form.
(0.03 MB DOC)

Text S4 Survey questions.
(0.02 MB DOC)
