# Supplementary figures and images for: Common HLA Alleles Associated with Health, but Not with Facial Attractiveness
Source: PLoS One. 2007 Jul 25;2(7):e640. doi: 10.1371/journal.pone.0000640 (PMC1919430; doi:10.1371/journal.pone.0000640)

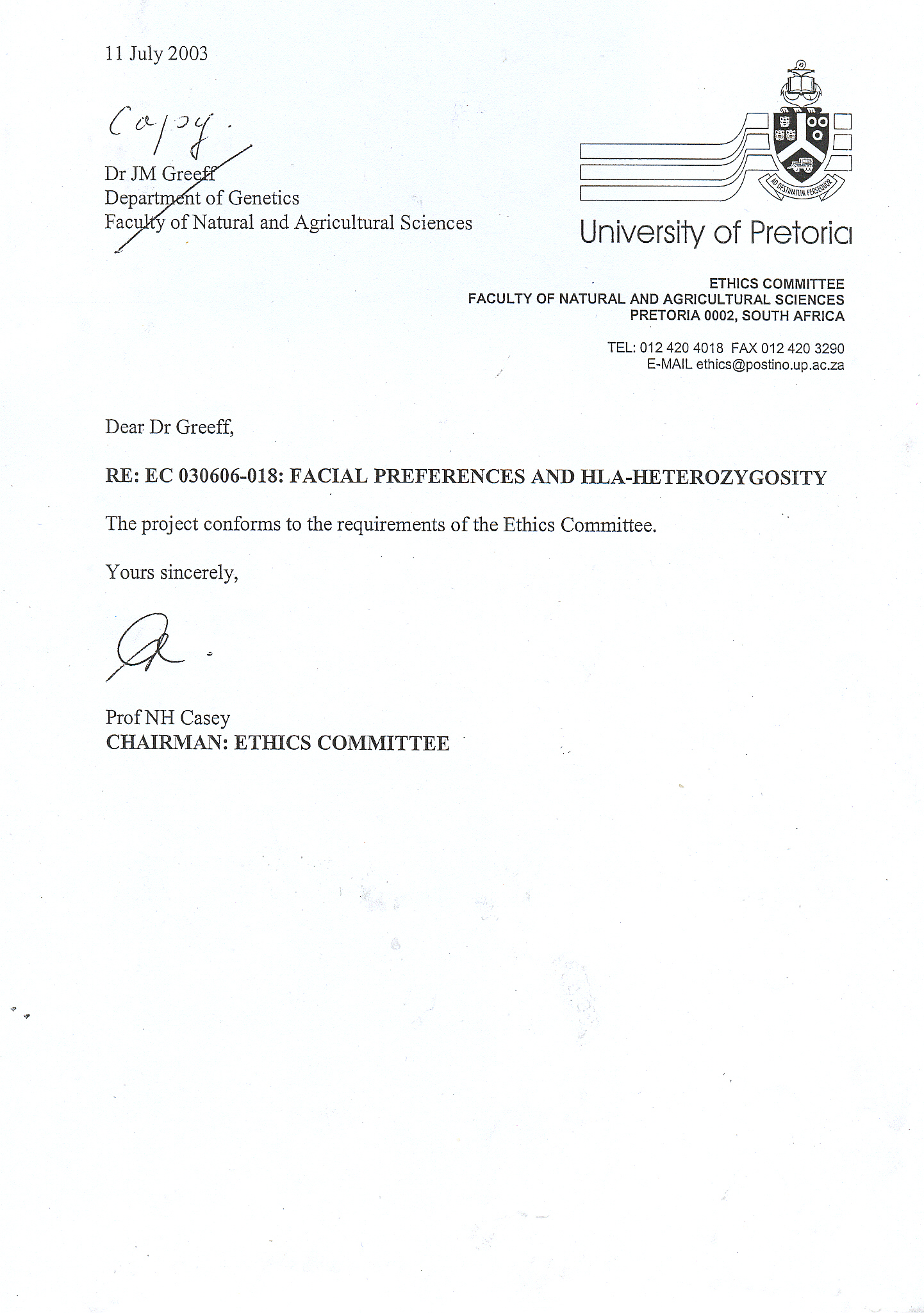

Supplement: Text S1 — Ethical approval-University of Pretoria. (1.22 MB JPG) [file pone.0000640.s001.jpg]

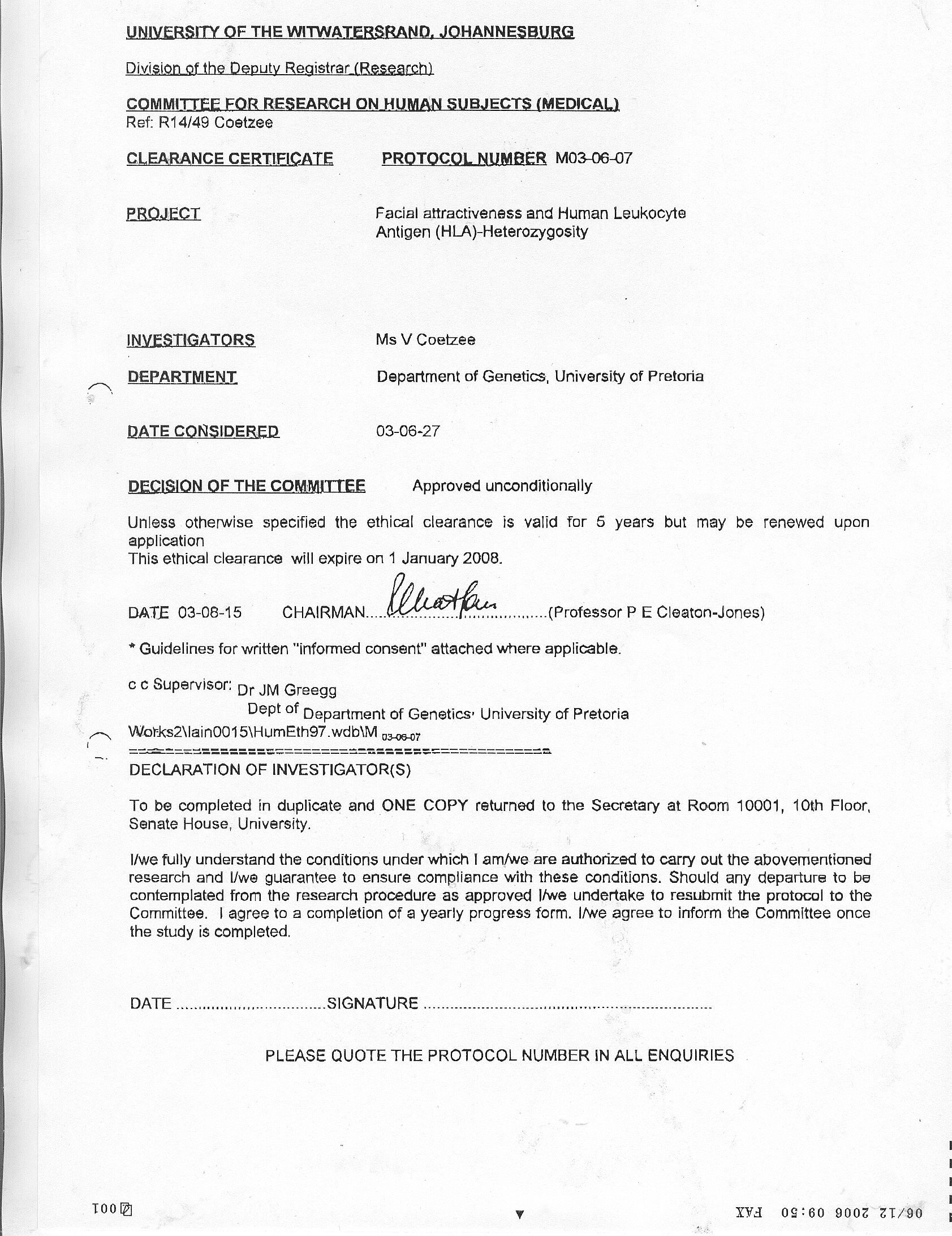

Supplement: Text S2 — Ethical approval-University of the Witwatersrand. (1.77 MB JPG) [file pone.0000640.s002.jpg]
