# Supplementary material for: Common HLA Alleles Associated with Health, but Not with Facial Attractiveness
Source: PLoS One. 2007 Jul 25;2(7):e640. doi: 10.1371/journal.pone.0000640 (PMC1919430; doi:10.1371/journal.pone.0000640)
Supplement: Text S3 — Subject information and consent form. (0.03 MB DOC) [file pone.0000640.s003.doc]

**Subject Information and Consent form**

For centuries people have been fascinated by the concept of beauty. We, and our ancestors, have spent much time and money to enhance our attractiveness. Is this just rules determined by society or is there a fundamental and biological underpinning to our concept of beauty? This research study aims to look at facial attractiveness, how it is determined and how it affects our lives. It will form the basis of my (Vinet Coetzee) MSc degree at the Department of Genetics, University of Pretoria.

We invite you to take part in this study. Your decision whether or not to partake is entirely your own, and you will not lose any previous benefits if you decide not to. There is no cost for participation in this study. If you decide to partake and are chosen for the study, you will receive a gift, consisting of merchandise from FNB, coffee and lunch. Although this is the only benefit you will receive, you have the opportunity to enhance knowledge on facial attractiveness.

This is a short explanation of what will be expected of you if you decide to take part in the study.

- You will be asked to complete a preliminary questionnaire. Please answer all the questions truthfully. Based on this questionnaire you will be selected for the study or not. Unfortunately, we cannot accommodate all volunteers, since some of you will fall outside the studies criteria.
- If you are chosen for the study, you will be asked to complete a questionnaire containing questions on health, behaviour and a few general questions. Additionally, a facial photo and measurements will be taken like weight and height and you will be asked for a mouth swab. To perform the mouth swab we simply wipe the inside of your mouth with a cytology brush. The questionnaire and measurements will require approximately 45 minutes of your time. None of these measurements will be painful and the risks are judged to be minimal. Nevertheless, you are welcome to leave at any time during the study. As long as you were chosen for the study, you cannot lose your gift if you decide to leave. You also have the right to have your data destroyed at any point during or after the study.
- Once the photos are taken you will be asked to judge the photos of the opposite sex, which will require approximately 15 minutes of your time. For the women this will take place on another scheduled day, while the men will judge photos on the same day. Unfortunately, the women cannot complete the questionnaire and judge the photos on a single day, because we first have to obtain the male photos.
- It is important to note that all your information will be strictly confidential unless disclosure is required by law. Your personal details will be linked to a research number. All the questionnaires, measurements and photos will only have the research number to identify them. No other person will have access to your personal information except me. Your name will not be used in the reporting of information in publications and conference presentations.
- At the conclusion of the study, subjects will be fully debriefed as to the aims of the study.
- A copy of the findings will also be made available upon request by contacting investigator, Vinet Coetzee, at 073 144 5251 or supervisor Dr. Jaco Greeff at 012 420 3260.

**Voluntary Consent by the Participant: Participation in this research project is completely voluntary, and your consent is required before you can participate in this research. Your identity will not be revealed to anyone whatsoever, and all reference to you will be of a generic nature. If significant new information related to this study becomes available and this information may affect your willingness to participate in this study, Vinet Coetzee or Dr. Jaco Greeff will notify you immediately. Any questions can be addressed to me (Vinet Coetzee) as this study forms the bases of my MSc degree at the University of Pretoria.**

**I _____________________________________ declare that:**

**ID number____________________________________________**

**Contact number_______________________________________**

1. **I have read this form (or it has been read to me) and I fully understand the nature of the study and my participation in it.**
2. **I have not been coerced into participating in the project and willingly yield personal information to the researchers, without recourse.**
3. **The researchers may use the data without prejudice, in a scientific context and the results may be published in a thesis and scientific journal and presented at scientific meetings.**
4. **All of my questions concerning this research have been answered. If I have any questions in the future about this study they will be answered by the investigator listed above or her supervisor.**
5. **A copy of this form has been given to me.**

Participant’s Signature____________________ Date________________

Witness’s Signature______________________ Date_________________
