# Supplementary material for: Common HLA Alleles Associated with Health, but Not with Facial Attractiveness
Source: PLoS One. 2007 Jul 25;2(7):e640. doi: 10.1371/journal.pone.0000640 (PMC1919430; doi:10.1371/journal.pone.0000640)
Supplement: Text S4 — Survey questions. (0.02 MB DOC) [file pone.0000640.s004.doc]

SURVEY QUESTIONS

*Initial male/ female questionnaire:*

1. Are you currently using the pill or injection as contraception?
2. Female questionnaire: Are you heterosexual? (Do you prefer men as your sexual partners)
3. How healthy are you in general?
4. How many times were you ill in the last year?
5. How many times per year do you get the flu?
6. How many times per year do you get a cold?

*Image scoring questionnaire:*

1. Please indicate how attractive you think this woman is.
2. Please indicate how healthy you think this woman is.
3. Do you know any of these women?
